# Is there a role for immune checkpoint blockade with ipilimumab in prostate cancer?

**DOI:** 10.1002/cam4.64

**Published:** 2013-02-24

**Authors:** Edward Cha, Eric J Small

**Affiliations:** Department of Medicine, University of CaliforniaSan Francisco, California

**Keywords:** Castration-resistant prostate cancer, checkpoint blockade, immune response, immunotherapy, ipilimumab

## Abstract

Treatment for advanced prostate cancer has and will continue to grow increasingly complex, owing to the introduction of multiple new therapeutic approaches with the potential to substantially improve outcomes for this disease. Agents that modulate the patient's immune system to fight prostate cancer – immunotherapeutics – are among the most exciting of these new approaches. The addition of antigen-specific immunotherapy to the treatment of castration-resistant prostate cancer (CRPC) has paved the way for additional research that seeks to augment the activity of the immune system itself. The monoclonal antibody ipilimumab, approved in over 40 countries to treat advanced melanoma and currently under phase 2 and 3 investigation in prostate cancer, is thought to act by augmenting immune responses to tumors through blockade of cytotoxic T-lymphocyte antigen 4, an inhibitory immune checkpoint molecule. Ipilimumab has been studied in seven phase 1 and 2 clinical trials that evaluated various doses, schedules, and combinations across the spectrum of patients with advanced prostate cancer. The CRPC studies of ipilimumab to date suggest that the agent is active in prostate cancer as monotherapy or in combination with radiotherapy, docetaxel, or other immunotherapeutics, and that the adverse event profile is as expected given the safety data in advanced melanoma. The ongoing phase 3 program will further characterize the risk/benefit profile of ipilimumab in chemotherapy-naïve and -pretreated CRPC.

## Rationale for Immunotherapy in Prostate Cancer

For men across the world, prostate cancer is the second most common cancer diagnosis and the sixth leading cause of cancer-related death 1. Most diagnoses (approximately 71%) occur in developed countries where routine prostate-specific antigen (PSA) screening is practiced; thus, the disease is frequently diagnosed in the asymptomatic stage 1,2. Even before the appearance of clinical symptoms, immune responses against prostate tumors are evidenced by intratumoral leukocyte infiltration and inflammatory pathway activation 3,4. Analyses of tissue samples from prostate tumors have revealed infiltrating leukocytes with a role in innate immunity (i.e., natural killer cells), as well as those with antigen-specific activity (i.e., effector and regulatory T cells), suggesting that the host immune system can mount a natural antitumor response that employs both the innate and adaptive branches 3,4. Persistence and progressive growth of the tumor in an immunocompetent environment suggests that, as has been shown for many solid tumors, at least some prostate cancer cells develop the ability to avoid or suppress the host immune response 4,5. This body of evidence supports the rationale for combating prostate cancer with immunotherapy, with the goal of promoting more effective tumor control by encouraging the host to mount an immunogenic response to prostate tumor cells 4,6.

Because tumor-associated antigens may not be adequately recognized by the immune system for a productive immune response to result 3,5, antigen-specific anticancer immunotherapy (tumor vaccines) is designed to enhance the immunogenicity of known tumor-associated antigens, with the goal of promoting a productive antitumor immune response. Tumor vaccines may or may not employ an adjuvant component to enhance the function of antigen presenting cells (APCs) and immune effectors such as T cells 7. In 2010, sipuleucel-T (PROVENGE®; Dendreon, Seattle, WA) garnered the first national regulatory approval (by the United States Food and Drug Administration [FDA]) of a tumor vaccine and is now indicated for treatment of castration-resistant prostate cancer (CRPC) in patients with asymptomatic or minimally symptomatic disease 8,9. For prostate cancer oncologists and urologists, this approval helped to validate immunotherapy as a treatment approach with the potential to provide significant clinical benefits, and several other tumor vaccines are now under investigation for CRPC.

Targeting specific tumor-associated antigens is only one therapeutic approach with the potential to tip the balance from immune tolerance to immune activation against the tumor. Some preclinical and clinical evidence suggests that the CRPC tumor cells or the surrounding microenvironment are capable of suppressing the activity of infiltrating T effector cells, leading to immune tolerance toward the tumor in spite of an antigen-specific immune response 7,10,11. With the aim of overcoming this tolerance and augmenting immune responses to CRPC tumors, cancer researchers are investigating inhibition of molecules or pathways that dampen immune responses (so-called immune checkpoint receptors), in an effort to modulate the immune system itself to fight cancer 4,6.

Interestingly, standards of care like androgen deprivation therapy, radiotherapy, and chemotherapy 12 have been observed to alter the immune milieu of prostate cancer or the host, although the underlying pathways are not completely understood 4,6,7,10,13. Preclinical and preliminary clinical evidence suggest that radiotherapy and/or chemotherapy may augment immunotherapy-induced immunologic responses on a molecular level, possibly through antigen release from dying tumor cells 14,15,16 or by direct modulation of immune effector molecules 17. This evidence for synergy supports the rationale for investigating multimodal therapies involving immunotherapy and other anticancer agents with potentially immunosupportive mechanisms of action. Whether a particular combination, timing, or schedule maximizes clinical benefit of immunotherapy-containing regimens is a subject of ongoing research.

## CTLA-4 Blockade in Prostate Cancer

**Figure 1:**
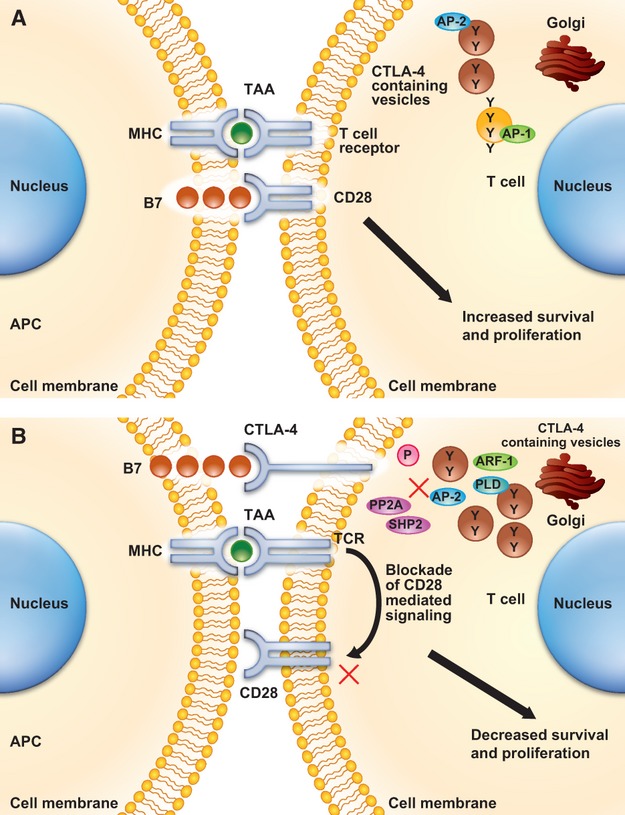
Role of CTLA-4 in T-cell activation and molecular consequence of CTLA-4 blockade. AP, adapter protein; APC, antigen presenting cell; ARF-1, ADP ribosylation factor 1; BCL-2, B cell lymphoma–associated protein 2; BCL-XL, B cell lymphoma–associated extra large protein; CTLA-4, cytotoxic T-lymphocyte antigen 4; MHC, major histocompatibility complex; NF-κB, nuclear factor kappa light-chain enhancer of activated B cells; P, phosphorylation (of the indicated target); PI3K, phosphoinositide 3 kinase; PLD, phospholipase D; PP2A, protein phosphatase 2A; SHP2, SH2 domain-containing protein tyrosine phosphatase 2; TAA, tumor-associated antigen. Reprinted from Salama A. K., Hodi F. S. Cytotoxic T-lymphocyte-associated antigen 4 18. Reprinted from *Clinical Cancer Research*, 2011, Vol. 17/Issue 14, 4622–4628, April K. S. Salama et al., “Is there a role for immune checkpoint blockage with ipilimumab in prostate cancer?”, with permission from AACR.

When APCs present antigens to T cells, the costimulatory interaction between APC-expressed B7 and T-cell–expressed CD28 is indispensable for a productive immune response to the antigen. To keep these responses in check, T-cell activation induces cell surface expression of immune checkpoint receptors, which has the net effect of downregulating T-cell activity 18–20. This process is thought to underlie the induction of peripheral tolerance. Cytotoxic T-lymphocyte antigen 4 (CTLA-4), the most extensively studied of these immune checkpoint receptors, binds to B7 with higher avidity than does CD28; thus, CTLA-4 competitively inhibits CD28-mediated T-cell activation and subsequently dampens the T-cell response (Fig. 1) 18. Blockade of CTLA-4 signaling preserves the CD28-B7 costimulatory signal and thus the T-cell activation signal, which augments the resulting T-cell-mediated immune responses.

Ipilimumab (YERVOY^TM^; Bristol-Myers Squibb, Princeton, NJ) is a fully human IgG_1_ monoclonal antibody against CTLA-4 21. Two randomized phase 3 trials of ipilimumab in advanced melanoma have reported overall survival benefit over control arms, with a side-effect profile that was managed with proactive treatment guidelines as outlined in the individual protocols 22–24. In 2011, the agent was approved in the United States as monotherapy at a dose of 3 mg/kg for treatment of unresectable or metastatic melanoma 24. The agent has since been approved as an advanced melanoma treatment in over 40 countries.

Due to its potentially broadly applicable mechanism of action, ipilimumab has also been under investigation in several other solid tumor settings, including CRPC. In fact, the first in-human study of ipilimumab was in prostate cancer: an investigation of a single 3 mg/kg dose of ipilimumab in 14 patients with hormone-refractory prostate cancer 25. Ipilimumab as a single dose had acceptable pharmacokinetic and safety profiles, but only two of the 14 patients experienced PSA declines of >50%. Results have since been reported for six other clinical studies of ipilimumab in CRPC. Together, these seven trials encompassed 240 patients across multiple settings of advanced CRPC, roughly 20% of whom had progressed on or relapsed after docetaxel (Table 1) 25–35.

**Table 1:** Summary of phase 1/2 data for ipilimumab in CRPC

Study/phase	Population (*n*)^1^	Regimen	Dose/schedule	Endpoints	Efficacy results^3^	Safety results^3^	Reference(s)
CA184-017^2^ NCT00323882 (Phase 2)	Metastatic CRPC, pre- and postdocetaxel (70)	Monotherapy or with one prior dose of XRT	3, 5, or 10 mg/kg monotherapy, 3 or 10 mg/kg with prior XRT, q3w × 4	Primary: safety Secondary: PSA responses, metabolic bone activity	21% (15 pts) had PSA responses (15.4% in chemo-naïve; 9.5% in chemo-pretreated)	11 pts with 13 grade ≥3 irAEs; no DLTs observed, no MTD or DLT	Slovin et al. 26
CA184-019^2^ NCT00050596 (Phase 2)	HRPC, docetaxel eligible (43)	Monotherapy or with docetaxel	3 mg/kg, monthly × 4	Safety and activity between arms	Two pts who received ipilimumab monotherapy and one pt who received ipilimumab + docetaxel had confirmed PSA responses	18 pts experienced 52 SAEs, 10 of which were attributed to ipilimumab	Small et al. 27
CA184-009^2^ (Phase 1)	Advanced HRPC; pre- and postchemo (14)	Monotherapy	3 mg/kg, one dose (two retreated at progression)	Safety, PK, PSA, objective responses (per reference at right)	Two pts had PSA declines of ≥50%	One pt experienced grade 3 rash/pruritus	Small et al. 25
CA184-118 NCT00170157 (Phase 2)	Advanced chemo-naïve CRPC (108)	With AA versus AA alone	3 mg/kg, one dose	Primary: percent without progression at 18 months Secondary: PSA responses	55% versus 38% undetectable PSA by 3 months	In combination arm, grade ≥3 irAEs included colitis (4.5%) and diarrhea (4.5%)	Tollefson et al. 28
CA184-098 NCT00064129 (Phase 1/2)	Metastatic CRPC, docetaxel eligible (24) (36)	With GM-CSF	Phase 1: 0.5, 1.5, 3, 5, or 10 mg/kg, monthly; expansion: 3 mg/kg monthly × 6	Primary for phase 1: MTD and safety; primary for expansion: PSA response Secondary for phase 1: T-cell immunity/response, PK, PSA response, ORR; secondary for expansion: percent activated naïve/memory T cells, epitopes for prostate antigens, T-cell response in HLA^+^ pts, safety, PSA response, ORR	At 3 mg/kg, 50% (three pts) had PSA declines of ≥50%, 1 PR; at 10 mg/kg, 17% (one pt) had PSA declines of ≥50%	irAEs correlated with dose (≥3 mg/kg) and PSA response	Small et al. 29; Fong et al. 30; Harzstark et al. 31
CA184-119 (Phase 1)	Metastatic chemo-naïve HRPC (12 escalation, 16 expansion)	With GVAX	0.3, 1, 3, or 5 mg/kg, monthly; expansion = 3 mg/kg	Primary: safety, MTD Secondary: TTP, PSA response, immune response, reduction in metabolic bone activity, survival	Six pts (five in escalation, one in expansion) receiving ≥3 mg/kg had PSA declines of ≥50%	Escalation cohort: five pts who received 3 or 5 mg/kg had grade ≥2 irAEs (four had hypophysitis, one had sarcoid alveolitis [DLT]; expansion cohort: two pts had grade 2 hypophysitis, three pts had grade 1 or 2 colitis, and one pt had grade 3 hepatitis	van den Eertwegh et al. 32
CA184-100 NCT00124670 (Phase 1)	Metastatic CRPC, chemo-naïve (24) and postdocetaxel (6)	With PSA-Tricom/PROSTVAC + GM-CSF	1, 3, 5, or 10 mg/kg, monthly × 6	Primary: MTD of regimen, safety Secondary: PSA and RECIST responses in HLA-A2^+^ pts; immunologic response (increase in PSA-specific T cells	15 pts had PSA declines (14 chemo-naïve, one pt postdocetaxel, all 3, 5, or 10 mg/kg), six were ≥50%; median PFS in chemo-naïve and postdocetaxel pts was 5.9 and 2.4 months; 3/12 unconfirmed PRs; median OS 34.4 months; 2-year OS 73%	No DLTs; most common AE was grade 1/2 site reaction; 8 (0/3, 2/6, 2/6 and 4/15 events in 1, 3, 5, or 10 mg/kg cohorts, respectively) grade ≥3 irAEs: 3 endocrinopathies, 2 rash, 1 diarrhea, 1 neutropenia, 1 elevated liver enzymes	Mohebtash et al. 33; Mohebtash et al. 34; Madan et al. 35

AA, androgen ablation; AE, adverse event; chemo, chemotherapy; CRPC, castration-resistant prostate cancer; DLT, dose-limiting toxicity; GM-CSF, granulocyte macrophage colony-stimulating growth factor; HLA, human leukocyte antigen; HRPC, hormone-refractory prostate cancer; irAEs, immune-related adverse events; MTD, maximum-tolerated dose; ORR, overall response rate; OS, overall survival; PFS, progression-free survival; PK, pharmacokinetics; PR, partial response; PSA, prostate-specific antigen; pt, patient; qXw, every X weeks; SAEs, serious (grade ≥3) adverse events; TTP, time to progression; XRT, radiotherapy.

1 Population descriptors are listed as defined in publication of data.

2 Trial sponsored by Bristol-Myers Squibb.

3 Results as reported in publicly available materials; most recent publicly available reports may be of interim data.

Reflecting the dose-finding nature of many of these trials, the studies utilized multiple doses (ranging from 0.5 to 10 mg/kg as either monotherapy or in combination studies) and schedules (ranging from a single dose of ipilimumab to recurring doses every 3 weeks for four cycles). In the phase 1 and 2 trials in CRPC, ipilimumab was active whether used as monotherapy 25,28 or with other interventions such as radiotherapy 26, docetaxel 27, or other immunotherapeutics with different mechanisms of action 29–35. Notably, one study in 43 chemotherapy-eligible patients compared ipilimumab monotherapy (3 mg/kg, every 4 weeks, for four doses) with ipilimumab plus a single 75 mg/m^2^ dose of docetaxel on day 1; this small study did not reveal a clear difference in safety or PSA response (around 15%) between the two groups 27.

Clinical investigation in CRPC with the antigen-specific therapies sipuleucel-T 9 and PROSTVAC® (Bavarian Nordic, Kvistgaard, Denmark) 36 revealed little overall impact on delaying progression of disease, but the reported benefits in overall survival may represent a slowing of tumor growth 7,26. In phase 2 reports of ipilimumab, although efficacy endpoints also varied amongst the studies, declines in PSA were consistently noted (15–20%) at doses of ≥3 mg/kg. All trials utilized the PSA Working Group 2 criteria 37. These response rates suggest that a subset of patients with CRPC may be responsive to therapy with ipilimumab, although none of the data reported to date have revealed specific characteristics of this subset. However, these studies did report various patterns of PSA declines, occurring at treatment onset, after a short period of stable disease, or within 6 months after an initial rise in PSA levels. Some immediate responses (within 1 month of initiating treatment) were noted, and some late responses (after 6 months of treatment) were also observed 26,27. In some cases, responses to ipilimumab lasted for 1 year or more 26,27. Although these phase 2 data require further verification in phase 3 trials, these observed patterns do suggest that an individual's immune response to cancerous cells may be dynamic and, in a select number of patients, durable over the course of disease.

In the CRPC trials, the side effects associated with ipilimumab therapy were common and inflammatory in nature (reflective of the immunologic mechanism of action). Table 2 provides incidences of these immune-related adverse events (irAEs) from the largest phase 2 trial, in which ipilimumab was used as monotherapy at 10 mg/kg (CA184-017) 26. Commonly reported side effects in the CRPC studies included gastrointestinal events such as colitis, diarrhea, rash or pruritus, hepatitis, or endocrinopathies. These endocrinopathies included adrenal insufficiency, hyper- or hypothyroidism, hypophysitis, or hypopituitarism. Interestingly, endocrinopathies leading to adrenally insufficient states (and suppressed androgen production) may also provide an indirect mechanism for tumor control, but as PSA responses to ipilimumab have been reported in the absence of changes in adrenal hormones 30, it is more likely that tumor responses are directly mediated by an ipilimumab-induced immune response. The phase 1 and 2 studies in CRPC did not report any safety issues that were unexpected given the safety data previously reported for ipilimumab 22,23. Though these side effects were severe in some instances, they were typically addressed through interventions outlined in the individual trial protocols, as was done in the melanoma studies. In study CA184-017, corticosteroids and other immunosuppressive agents, as well as hormone replacement therapy for endocrine side effects, were utilized at the discretion of the investigators 26.

**Table 2:** Common treatment-related adverse events observed with ipilimumab monotherapy at 10 mg/kg in a phase 2 study 26

	Ipilimumab –XRT *n* = 16 (%)	Ipilimumab +XRT *n* = 34 (%)	Ipilimumab overall (±XRT) *n* = 50 (%)
Any treatment-related AE			
Any Grade	16 (100)	29 (85)	45 (90)
Grade 3	7 (44)	13 (38)	20 (40)
Grade 4	3 (19)	0	3 (6)
Any immune-related AE (irAE)			
Any Grade	16 (100)	29 (85)	45 (90)
Grade 3	7 (44)	13 (38)	20 (40)
Grade 4	3 (19)	0	3 (6)
Common^1^ irAEs: any grade; Grade 3			
Colitis	7 (44); 6 (38)	4 (12); 2 (6)	11 (22); 8 (16)
Diarrhea	13 (81); 2(13)	14 (41); 2 (6)	27 (54); 4 (8)
Rash	9 (56); 0	7 (21); 0	16 (32); 0
Pruritus	6 (38); 1 (6)	4 (12); 0	10 (20); 1 (2)
Common^7^ laboratory abnormalities^2^: any grade; Grade 3; Grade 4			
Evaluable patients	15	34	49
Hemoglobin	12 (80); 1 (7); 0	28 (82); 6 (18); 0	40 (82); 7 (14); 0
Lymphocytes	12 (80); 2 (13); 0	31 (91); 3 (9); 0	43 (88); 5 (10); 0
ALT	7 (47); 1 (7); 1 (7)	10 (29); 1 (3); 0	17 (35); 2 (4); 1 (2)
AST	6 (40); 1 (7); 1 (7)	8 (24); 0; 0	14 (29); 1 (2); 1 (2)
AP	7 (47); 1 (7); 0	21 (62); 4 (12); 1 (3)	28 (57); 5 (10); 1 (2)
Amylase	4 (27); 0; 0	4 (12); 1 (3); 0	8 (16); 1 (2); 0

XRT, radiotherapy; ALT, alanine aminotransferase; AST, aspartate aminotransferase; AP, alkaline phosphatase; irAE, immune-related adverse event. Data from Slovin et al. 26.

1 Defined as AE or laboratory abnormality of any grade in ≥15% of patients in the 10 mg/kg ± XRT group.

2 Calculated from laboratory values.

Only one of the phase 2 studies in CRPC reported a dose-limiting toxicity (one case of grade 4 sarcoid alveolitis in a patient who received 5 mg/kg of ipilimumab and GVAX) 35, but this has not been recapitulated in other studies of ipilimumab monotherapy or combination therapy at 10 mg/kg 26,29–30,33–35. Phase 2 dose-ranging studies for ipilimumab in advanced melanoma suggested that the risk/benefit profile was more favorable at 10 mg/kg than it was for 3 mg/kg 16. Although it is not known whether these results are translatable to prostate cancer, when taken together, the reports in melanoma, and the overall tolerability of the 10-mg/kg dose in CRPC trials where it was evaluated, suggest that 10 mg/kg is appropriate for further study of ipilimumab in CRPC.

## Open Questions, Ongoing Trials, and Future Directions

**Figure 2:**
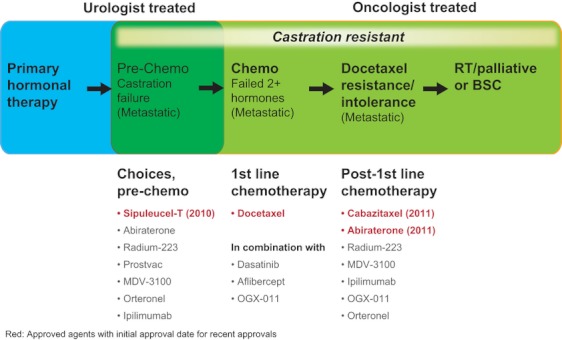
Anticancer agents US- or EU-approved or under phase 3 investigation for castration-resistant prostate cancer (CRPC). Data from http://www.fda.gov 38, http://www.ema.europa.eu [Bibr b39], and http://www.clinical.trials.gov 40.

Ongoing research in prostate cancer has broadened, and it is continuing to augment the therapeutic choices available to treat this disease. Immunotherapy is a promising but relatively recent addition, and while the concept of immunotherapy in CRPC was solidified with the US approval of sipuleucel-T, the ideal fit for immunotherapy in the CRPC treatment paradigm is a matter of continued study (Fig. 2) 38–40.

In an effort to understand in which settings ipilimumab might provide benefit in CRPC, it is under phase 2 and 3 investigation in both the chemotherapy-naïve and -pretreated settings (Table 3). In addition, other antigen-specific approaches are the subjects of ongoing clinical investigation (reviewed in Cha 7). It is hoped that these studies will help answer some of the questions that the medical community has regarding immunotherapy in CRPC.

**Table 3:** Summary of ipilimumab clinical trials in CRPC

Study	Phase/setting	Design [Primary endpoint]	Site(s)
NCT01057810	Phase 3 1st line CRPC	Ipilimumab (10 mg/kg q3w × 4 → q12w) versus placebo [OS]	International
NCT00861614	Phase 3 2nd+ line CRPC	Single-dose XRT → randomization to ipilimumab (10 mg/kg q3w × 4 → q12w) versus placebo [OS]	International
NCT01194271	Phase 2 Neoadjuvant	Ipilimumab (10 mg/kg q3w × 3) + hormone ablation → radical prostatectomy [safety]	US
NCT01377389	Phase 2 1st line HS	Ipilimumab (10 mg/kg q4w × 4) + leuprolide acetate (7.5 mg/month × 8) [response by PSA]	US
NCT00170157	Phase 2 1st line HS	Ipilimumab (3 mg/kg once) + leuprolide (7.5 mg/month × 3) + bicalutamide (50 mg/day × 3 months) versus leuprolide + bicalutamide alone [PFS]	US
NCT01530984	Phase 2 1st line CRPC	Ipilimumab (10 mg/kg q4w × 6) + GM-CSF (250 mcg/m^2^ days 1–14 × 6) versus ipilimumab alone [response by PSA]	US
NCT00064129	Phase 1 1st line CRPC	Ipilimumab (0.5–3 mg/kg q4w) + GM-CSF (250 mcg/m^2^ days 1–14 × 4) [MTD, safety, reduction in PSA]	US

CRPC, castration-resistant prostate cancer; GM-CSF, granulocyte macrophage colony-stimulating growth factor; HS, hormone sensitive; MTD, maximum-tolerated dose; OS, overall survival; PFS, progression-free survival; PSA, prostate-specific antigen; qXw, every X weeks; XRT, radiotherapy.

Treating physicians must always consider the question of risk/benefit when considering a therapeutic approach, and for immunotherapy, more data is needed to firmly address this issue. In the case of ipilimumab, phase 2 studies in CRPC suggest that PSA declines may occur in roughly 15–20% of patients. However, in the phase 3 trials of ipilimumab in melanoma, some responses to ipilimumab therapy initially appeared as disease progression (e.g., increases in total tumor burden or number of lesions) 16,22,23. If the response patterns seen in the phase 3 studies are suggestive of what can be expected in CRPC, it is possible that in some cases the benefit with ipilimumab may not be readily apparent for several weeks or months. It is for this reason that the phase 3 clinical trials of ipilimumab specify confirmation of disease progression 41,42.

The experience with irAEs associated with ipilimumab therapy in CRPC is limited to phase 1 and 2 data, but the agent's clinical development in melanoma has provided more extensive characterization of side effects and how to proactively manage them 22,23. Prompt treatment with corticosteroids or other immunosuppressive therapy, skipping a dose of ipilimumab, or in some cases discontinuation of ipilimumab therapy permitted resolution of the majority of severe events in a matter of days to weeks 22. It was also noted that prophylactic budesonide does not reduce the incidence of grade ≥2 diarrhea during therapy with ipilimumab for advanced melanoma 17. In the phase 1/2 reports for CRPC, ipilimumab's side-effect profile did not greatly differ from that reported for melanoma, but there is always a potential for patients with CRPC to experience side effect incidence or severity not anticipated from experience in other tumor types; for example, pelvic irradiation in older patients with CRPC might predispose them to side effects such as colitis. The phase 3 clinical trials in CRPC will help answer these questions and clarify whether the current side effect management guidelines, established in the phase 3 melanoma trials, will benefit patients with CRPC.

Finally, even though the phase 2 data in CRPC supports the use of 10 mg/kg as the phase 3 investigational dose, it is not yet clear whether this dose provides the ideal risk/benefit profile for CRPC. The phase 3 trials in CRPC will not utilize doses lower than 10 mg/kg, and it is uncertain what the risk/benefit profile of a lower dose would be.

Physicians must also consider whether a given therapy is the right choice for an individual patient. Currently, the prostate cancer community does not know to what extent different types of immunotherapy will be best suited for different subsets of patients, as heretofore no clinical or molecular parameters have clearly emerged as biomarkers. Encouragingly, however, studies of ipilimumab and ipilimumab-based combination therapy in melanoma and CRPC have identified new leads. Antibody and T-cell responses to NY-ESO-1 43, high baseline expression of immune markers such as FoxP3 and indoleamine 2,3-dioxygenase 44, increases in tumor-infiltrating lymphocytes, and changes in expression of immune-related genes 44 have all been correlated with clinical activity or benefit of ipilimumab. High baseline frequency of differentiated CD8^+^ T cells, high baseline frequency of CD4^+^ T cells that express CTLA-4 or PD-1, and low pretreatment frequencies of differentiated CD4^+^ or regulatory T cells have been associated with significantly longer survival following ipilimumab therapy 45. Furthermore, analysis of CD4^+^ and CD8^+^ T cells from ipilimumab-treated patients correlated low baseline expression of Ki67, a marker for T-cell proliferation, and eomesodermin, a transcription factor associated with effector T-cell function, with relapse or irAEs from ipilimumab therapy 46. Ongoing ipilimumab studies in multiple tumor types are evaluating various clinical and molecular parameters as biomarkers for survival or other clinical benefit, safety, or immunologic competence.

Chronic use of immunosuppressive corticosteroids, a therapeutic option among solid tumors unique to prostate cancer, may also be detrimental to ipilimumab's activity, and sustaining an active and durable immune response after ipilimumab treatment may benefit from delaying the initiation of corticosteroid-heavy regimens. There is currently no appropriate analysis to determine to what extent prolonged corticosteroid use and its sequence in CRPC treatment affect ipilimumab activity.

As mentioned above, their nonoverlapping mechanisms of action suggest the potential for combination therapy between multiple immunotherapy approaches, particularly antigen-specific therapies such as sipuleucel-T and antigen-independent therapies such as ipilimumab. To further explore the utility of such combinations, phase 1 or 2 studies pairing ipilimumab in regimens with sipuleucel-T, off-the-shelf prostate cancer vaccines, and/or adjuvants such as granulocyte macrophage colony-stimulating growth factor (GM-CSF) are complete or underway 31,32. One recent study, a phase 1 dose escalation trial of ipilimumab given concurrently with a fixed dose of GM-CSF–transduced allogeneic prostate cancer cells, reported that the higher doses (3 mg/kg and 5 mg/kg) were tolerated well in most patients and produced some PSA declines of >50% in seven patients (25% of patients in the 3 mg/kg or 5 mg/kg cohorts) 32. Although these trials have not and will not directly address how the sequence of therapies may affect results, as data from these studies and the phase 3 trials mature, clarification of issues above will further our understanding of the ideal placement for ipilimumab, and possibly other immunotherapy, into the CRPC treatment paradigm.

## References

[b1] GLOBOCAN (2008). Prostate cancer incidence and mortality worldwide in 2008 summary. http://globocan.iarc.fr/factsheets/cancers/prostate.asp.

[b2] American Cancer Society Global cancer facts & figures 2nd edition. http://www.cancer.org/acs/groups/content/@epidemiologysurveilance/documents/document/acspc-027766.pdf.

[b3] Diener KR, Woods AE, Manavis J, Brown MP, Hayball JD (2009). Transforming growth factor-beta-mediated signaling in T lymphocytes impacts on prostate-specific immunity and early prostate tumor progression. Lab. Invest.

[b4] May KF, Gulley JL, Drake CG, Dranoff G, Kantoff PW (2011). Prostate cancer immunotherapy. Clin. Cancer Res.

[b5] Degl'Innocenti E, Grioni M, Boni A (2005). Peripheral T cell tolerance occurs early during spontaneous prostate cancer development and can be rescued by dendritic cell immunization. Eur. J. Immunol.

[b6] Drake CG (2010). Prostate cancer as a model for tumour immunotherapy. Nat. Rev. Immunol.

[b7] Cha E, Fong L (2011). Immunotherapy for prostate cancer: biology and therapeutic approaches. J. Clin. Oncol.

[b8] (2010). PROVENGE® (sipuleucel-T) [US package insert]..

[b9] Kantoff P, Higano CS, Shore ND (2010). Sipuleucel-T immunotherapy for castration-resistant prostate cancer. N. Engl. J. Med.

[b10] Gannon PO, Poisson AO, Delvoye N, Lapointe R, Mes-Masson AM, Saad F (2009). Characterization of the intra-prostatic immune cell infiltration in androgen-deprived prostate cancer patients. J. Immunol. Methods.

[b11] Kiniwa Y, Miyahara Y, Wang HY (2007). CD8+ Foxp3+ regulatory T cells mediate immunosuppression in prostate cancer. Clin. Cancer Res.

[b12] National Comprehensive Cancer Network NCCN clinical practice guidelines in oncology–prostate cancer. V.3.2010. http://www.nccn.org.

[b13] Aragon-Ching JB, Williams KM, Gulley JL (2007). Impact of androgen-deprivation therapy on the immune system: implications for combination therapy of prostate cancer. Front. Biosci.

[b14] Apetoh L, Ghiringhelli F, Tesnière A (2007). Toll-like receptor 4–dependent contribution of the immune system to anticancer chemotherapy and radiotherapy. Nat. Med.

[b15] Tesnière A, Apetoh L, Ghiringhelli F (2008). Immunogenic cancer cell death: a key-lock paradigm. Curr. Opin. Immunol.

[b16] Wolchok JD, Neyns B, Linette G (2010). Ipilimumab monotherapy in patients with pretreated advanced melanoma: a randomised, double-blind, multicentre, phase 2, dose-ranging study. Lancet Oncol.

[b17] Weber J, Thompson JA, Hamid O (2009). A randomized, double-blind, placebo-controlled, phase II study comparing the tolerability and efficacy of ipilimumab administered with or without prophylactic budesonide in patients with unresectable stage III or IV melanoma. Clin. Cancer Res.

[b18] Salama AK, Hodi FS (2011). Cytotoxic T-lymphocyte-associated antigen-4. Clin. Cancer Res.

[b19] Melero I, Hervas-Stubbs S, Glennie M, Pardoll DM, Chen L (2007). Immunostimulatory monoclonal antibodies for cancer therapy. Nat. Rev. Cancer.

[b20] de la Cruz-Merino L, Grande-Pulido E, Albero-Tamarit A, Codes-Manuel de Villena ME (2008). Cancer and immune response: old and new evidence for future challenges. Oncologist.

[b21] Hoos A, Ibrahim R, Korman A (2010). Development of ipilimumab: contribution to a new paradigm for cancer immunotherapy. Semin. Oncol.

[b22] Hodi FS, O'Day SJ, McDermott DF (2010). Improved survival with ipilimumab in patients with metastatic melanoma. N. Engl. J. Med.

[b23] Robert C, Thomas L, Bondarenko I (2011). Ipilimumab plus dacarbazine for previously untreated metastatic melanoma. N. Engl. J. Med.

[b24] YERVOY™ (ipilimumab) [US package insert] (2011).

[b25] Small EJ, Tchekmedyian NS, Rini BI, Fong L, Lowy I, Allison JP (2007). A pilot trial of CTLA-4 blockade with human anti-CTLA-4 in patients with hormone-refractory prostate cancer. Clin. Cancer Res.

[b26] Slovin SF, Hamid O, Tejwani S (2012). Ipilimumab (IPI) in metastatic castrate-resistant prostate cancer (mCRPC): results from an open-label, multicenter phase I/II study. J. Clin. Oncol.

[b27] Small E, Higano C, Tchekmedyian N (2006). Randomized phase II study comparing 4 monthly doses of ipilimumab (MDX-010) as a single agent or in combination with a single dose of docetaxel in patients with hormone-refractory prostate cancer.

[b28] Tollefson MK, Karnes RJ, Thompson RH (2010). A randomized phase II study of ipilimumab with androgen ablation compared with androgen ablation alone in patients with advanced prostate cancer.

[b29] Small EJ, Weinberg V, Kavanaugh B, Valiente J, Rini BI, Fong L (2007). Combination immunotherapy with GM-CSF and ipilimumab (anti-CTLA4 antibody) in patients with metastatic hormone refractory prostate cancer.

[b30] Fong L, Kwek SS, O'Brien S (2009). Potentiating endogenous antitumor immunity to prostate cancer through combination immunotherapy with CTLA4 blockade and GM-CSF. Cancer Res.

[b31] Harstark AL, Fong L, Weinberg VK (2010). Final results of a phase I study of CTLA-4 blockade in combination with GM-CSF for metastatic castration resistant prostate cancer (mCRPC). J. Clin. Oncol.

[b32] van den Eertwegh AJ, Versluis J, van den Berg HP (2012). Combined immunotherapy with granulocyte-macrophage colony-stimulating factor-transduced allogeneic prostate cancer cells and ipilimumab in patients with metastatic castration-resistant prostate cancer: a phase 1 dose-escalation trial. Lancet Oncol.

[b33] Mohebtash M, Madan RA, Arlen PM (2009). Phase I trial of targeted therapy with PSA-TRICOM vaccine (V) and ipilimumab (ipi) in patients (pts) with metastatic castration-resistant prostate cancer (mCRPC). J. Clin. Oncol.

[b34] Mohebtash M, Madan RA, Rauckhorst M (2009). Phase I trial of PSA-TRICOM vaccine and ipilimumab in patients (Pts) with metastatic castrate-resistant prostate cancer (mCRPC).

[b35] Madan RA, Mohebtash M, Arlen PM (2012). Ipilimumab and a poxviral vaccine targeting prostate-specific antigen in metastatic castration-resistant prostate cancer: a phase 1 dose-escalation trial. Lancet Oncol.

[b36] Kantoff PW, Schuetz TJ, Blumenstein BA (2010). Overall survival analysis of a phase II randomized controlled trial of a poxviral-based PSA-targeted immunotherapy in metastatic castration-resistant prostate cancer. J. Clin. Oncol.

[b37] Arlen PM, Bianco F, Dahut W (2008). Prostate specific antigen working group guidelines on prostate specific antigen doubling time. J. Urol.

[b38] US Food and Drug Administration Drugs. http://www.fda.gov.

[b39] European Medicines Agency http://www.ema.europa.eu.

[b40] US National Institutes of Health http://www.clinicaltrials.gov.

[b41] Beer TM, Logothetis C, Sharma P (2011). Randomized, double-blind, phase III trial to compare the efficacy of ipilimumab (Ipi) versus placebo in asymptomatic or minimally symptomatic patients (pts) with metastatic chemotherapy-naïve castration-resistant prostate cancer (CRPC). J. Clin. Oncol.

[b42] Drake CG, Scher HI, Gerritsen W (2011). A randomized, double-blind, phase III trial comparing ipilimumab versus placebo following radiotherapy (RT) in patients (pts) with castration-resistant prostate cancer (CRPC) who have received prior treatment with docetaxel (D). J. Clin. Oncol.

[b43] Yuan J, Adamow M, Ginsberg BA (2011). A prospective phase II trial exploring the association between tumor microenvironment biomarkers and clinical activity of ipilimumab in advanced melanoma. J. Transl. Med.

[b44] Hamid O, Schmidt H, Nissan A (2011). A prospective phase II trial exploring the association between tumor microenvironment biomarkers and clinical activity of ipilimumab in advanced melanoma. J. Transl. Med.

[b45] Santegoets SJ, Stam AG, Lougheed SM (2012). T cell profiling reveals high CD4(+)CTLA-4 (+) T cell frequency as dominant predictor for survival after Prostate GVAX/ipilimumab treatment. Cancer Immunol. Immunother.

[b46] Wang W, Yu D, Sarnaik AA (2012). Biomarkers on melanoma patient T cells associated with ipilimumab treatment. J. Transl. Med.

